# Impaired frequencies and function of platelets and tissue remodeling in chronic Chagas disease

**DOI:** 10.1371/journal.pone.0218260

**Published:** 2019-06-14

**Authors:** Claudia Pengue, Gonzalo Cesar, María Gabriela Alvarez, Graciela Bertocchi, Bruno Lococo, Rodolfo Viotti, María Ailén Natale, Melisa D. Castro Eiro, Silvia S. Cambiazzo, Nancy Perroni, Myriam Nuñez, María Cecilia Albareda, Susana A. Laucella

**Affiliations:** 1 Hospital Interzonal General de Agudos Eva Perón, Buenos Aires, Argentina; 2 Instituto Nacional de Parasitología Dr. M. Fatala Chaben, Buenos Aires, Argentina; 3 Hospital General de Agudos Dr. Teodoro Álvarez, Buenos Aires, Argentina; 4 Departamento de Matemática y Física, Facultad Farmacia y Bioquímica, Universidad de Buenos Aires, Argentina; Institut d'Investigacions Biomediques de Barcelona, SPAIN

## Abstract

Chronic inflammation, as a consequence of the persistent infection with *Trypanosoma cruzi*, leads to continuous activation of the immune system in patients with chronic Chagas disease. We have previously shown that increased sera levels of soluble P-selectin are associated with the severity of the cardiomyopathy distinctive of chronic Chagas disease. In this study, we explored the expression of biomarkers of platelet and endothelial activation, tissue remodeling, and mediators of the coagulation cascade in patients at different clinical stages of chronic Chagas heart disease. The frequencies of activated platelets, measured by the expression of CD41a and CD62P were decreased in patients with chronic Chagas disease compared with those in uninfected subjects, with an inverse association with disease severity. Platelet activation in response to adenosine diphosphate was also decreased in *T*. *cruzi*-infected subjects. A major proportion of *T*. *cruzi* infected subjects showed increased serum levels of fibrinogen. Patients with severe cardiac dysfunction showed increased levels of endothelin-1 and normal values of procollagen I. In conclusion, chronic infection with *T*. *cruzi* induced hemostatic alterations, even in those patients who do not yet present cardiac symptoms.

## Introduction

*Trypanosoma cruzi*, the causative agent of Chagas disease, infects 6–7 million people in Central and South America, as well as in countries historically nonendemic for *T*. *cruzi* infection [1]. The acute phase is characterized by the presence of a large number of parasites in the circulation and even though the immune response is able to control the infection, the parasite can survive establishing a chronic infection.

Chronic inflammation, as a consequence of the persistent infection with *T*. *cruzi*, leads to continuous activation of the immune system in chronic Chagas disease patients [2–5]. Inflammatory mediators regulate the expression of different adhesion molecules that participate in the recruitment of leukocytes and monocytes to sites of infection [6]. Among the latter group, platelet selectin (P-selectin) redistributes to the surface of platelets and endothelial cells within minutes after activation [7–9], and the P-selectin glycoprotein ligand-1 (PSGL-1) is constitutively expressed and participates in both leukocyte recruitment and the formation of platelet thrombi [10].

We have previously shown that increased serum levels of soluble P-selectin are associated with the severity of the cardiomyopathy that is distinctive of chronic Chagas disease [3]. However, children in early stages of *T*. *cruzi* infection also displayed high s-P-selectin titers, and these levels decreased following treatment with benznidazole [11]. Although increased s-P-selectin levels in chronic *T*. *cruzi* infection probably reflect alterations in the microcirculation that might eventually result in a pathogenic mechanism, it can also be reflective of an ongoing immune response to keep the parasite under control [3,12–15]. Of note, it is becoming more evident that platelets have inflammatory functions and can influence both innate and adaptive immune responses [16–18]. Here, we explored the expression of platelet and endothelial activation, tissue remodeling, and mediators of the coagulation cascade in patients at different clinical stages of chronic Chagas heart disease. The findings reported in the present work show platelet dysfunction and alterations in hemostatic factors in chronic Chagas disease.

## Materials and methods

### Selection of the study population

Subjects were recruited at the Chagas disease Section, Cardiology Department, Hospital Interzonal General de Agudos “Eva Perón”, Buenos Aires, Argentina. A positive *T*. *cruzi* infection was determined by indirect immunofluorescence assay, hemagglutination, and enzyme-linked immunoassay techniques [19]. Subjects testing positive in at least two of these tests were considered to be infected. Chronically *T*. *cruzi*-infected subjects were evaluated clinically and stratified according to a modified version of the Kuschnir grading system, as follows [20,21]. Group 0 (G0) had normal electrocardiograph (ECG), chest radiograph, and echocardiograph findings; Group 1 (G1) had normal chest radiograph and echocardiograph findings but abnormal electrocardiograph findings; Group 2 (G2) had ECG abnormalities and heart enlargement as determined by chest X-ray; and Group 3 (G3) had ECG abnormalities, heart enlargement and clinical or radiologic evidence of heart failure. For each experiment, age-matched uninfected healthy subjects were included as controls (Table 1). A group of patients suffering from dilated cardiomyopathy of noninfectious origin was also evaluated as a control group (Table 1). The inclusion criteria for heart failure (HF) patients were class I/II/III classification (New York Heart Association classification), with an ejection fraction of < 40% by echocardiography. The etiology for heart failure was hypertension in three patients, postchemotherapy in one patient, alcoholism in one patient and idiopathic dilated cardiomyopathy in four patients.

**Table 1:** Baseline characteristics of the study population.

Clinical stage	n	Age range	Sex	
		(median years)	Female	Male
Uninfected healthy subjects	35	18–76 (44)	20	15
G0	20	19–72 (42)	11	9
G1	16	25–67 (51.5)	7	9
G2	2	50–59 (54.5)	1	1
G3	14	42–76 (59)	3	11
HF^A^	9	34–81 (61)	1	8

Note.

^A^ HF, Patients with heart failure not related to Chagas disease in a compensated state.

This protocol was approved by the Institutional Review Boards of the Hospital Interzonal General de Agudos Eva Perón, Buenos Aires, Argentina. Patients with Chagas disease or uninfected controls with a history of hypertension, vascular, ischemic or congenital heart disease, cancer, HIV infection, syphilis, diabetes, arthritis or allergy, and with coagulation disorders were excluded from the study. Signed informed consent was obtained from all individuals prior to inclusion in the study.

### Collection of plasma and serum specimens

Five milliliters of blood was collected by venipuncture into tubes containing sodium citrate (Vacutainer; Becton Dickinson). To obtain plasma, blood was centrifuged at 1400 *g* for 20 min at 25°C. Blood to be used for serum analysis was allowed to coagulate at 4°C and centrifuged at 1000 *g* for 15 min for sera separation. Samples were stored at -80°C until use. Due to sample availability, assays were not run for all the samples.

### Detection of P-selectin (CD62P), CD63 and PSGL-1 expression in whole blood

Monoclonal antibodies were all purchased from Becton Dickinson. To measure the expression of P-selectin and CD63 in platelets, 5 μl of whole blood collected in citrate-containing tubes was diluted with 50 μl of PBS solution and stained with anti-CD41a (FITC-conjugated) and anti-CD62P (PE-conjugated) or anti-CD63 (PE-conjugated) monoclonal antibodies at room temperature in the dark for 20 min. Unstained samples were used as negative controls. Blood samples were fixed with 1% paraformaldehyde at 4°C for 30 min and analyzed on a FACSCalibur flow cytometer using CellQuest software (Becton Dickinson). Platelets were identified by side scatter and anti-CD41a-FITC immunofluorescence on a logarithmic-scaled dot plot. Ten thousand events gated on CD41a^+^ cells were collected per sample. Data are shown as the percentage of CD41a^+^ cells that express CD62 or CD63. For expression of PSGL-1 (CD162), 100 μl of whole blood was stained with anti-CD4 (PerCP-conjugated), anti-CD8 (FITC-conjugated) and anti-CD162 (PE-conjugated) monoclonal antibodies at room temperature in dark for 20 min. Subsequently, a FACS lysing solution (Becton Dickinson) was added, followed by two wash steps and fixation with 1% paraformaldehyde. Two hundred thousand events were collected. For analysis, lymphocytes were gated by forward and side-scatter parameters, and the percentage of CD4^+^CD162^+^ or CD8^+^CD162^+^ cells were calculated for each patient.

### Measurements of mediators of the coagulation cascade and endothelial activation

The platelet counts were measured using optical automated hematological counter Cell-Dyn Ruby (Abbot). Fibrinogen was measured by the Clauss technique using the STA-Liquid Fib reagents (Stago) [22], whereas the von Willebrand factor was determined by a turbidimetric method using the commercial kit LIATEST-VWF:Ag (Stago). Controls and calibrators for fibrinogen and von Willebrand measurement were from Stago and samples were analyzed with an automated coagulation analyzer STA Compact (Stago). Plasma levels of endothelin-1 (R & D Systems) and serum levels of procollagen I C-terminal propeptide (Takara Bio Inc.) were measured by capture ELISA using commercial kits and according to the manufacturer’s instructions.

### Measurement of platelet activation and platelet-leukocyte aggregation

Five microliters of whole blood was incubated with adenosine diphosphate (ADP) at a final concentration of 20 μmol/L at room temperature for 5 min. A control incubation was performed similarly, except that ADP was omitted. Then, samples were stained with anti-CD41a (FITC-conjugated) and anti-CD62P (PE-conjugated) monoclonal antibodies at room temperature in dark for 20 min. Blood samples were fixed with 1% paraformaldehyde at 4°C for 30 min and analyzed on a FACSCalibur flow cytometer using CellQuest software (Becton Dickinson). Platelets were identified by side scatter and anti-CD41a-FITC immunofluorescence on a logarithmic-scaled dot plot. Ten thousand events gated on CD41a^+^ cells were collected per sample. Data are shown as the percentage of CD41a^+^ cells that express CD62.For platelet-leukocyte aggregation assays, 50 microliters of whole blood was incubated with anti-CD41a (PE-conjugated) and anti-CD45 (FITC-conjugated) monoclonal antibodies at room temperature in dark for 20 min. Next, samples were fixed with 1% paraformaldehyde at 4°C for 30 min and analyzed on a FACSCalibur flow cytometer using CellQuest software (Becton Dickinson) [23,24]. Lymphocytes were identified based on cell size and granularity using the forward and side scatter. The analysis of CD45-FITC vs. CD41a-PE allowed the discrimination of platelet-coupled and platelet-free lymphocytes. Data are shown as the percentage of platelet-coupled lymphocytes in the lymphocyte population.

### Statistical analyses

Normality of the variables distribution was assessed using the Kolmogorov-Smirnov criterion. Differences between *T*. *cruzi*-infected and uninfected subjects were evaluated by Mann-Whitney U test or Student’s *t* test (two-tailed). Differences among groups were evaluated by ANOVA followed by a Bonferroni’s test for multiple comparisons or by a test for lineal trends. Correlation between variables was performed with the Pearson test. Differences were considered statistically significant when P values were < 0.05.

## Results

### Quantification of activated platelets and the counter-receptor on T cells in patients with chronic Chagas disease

**Fig 1:**
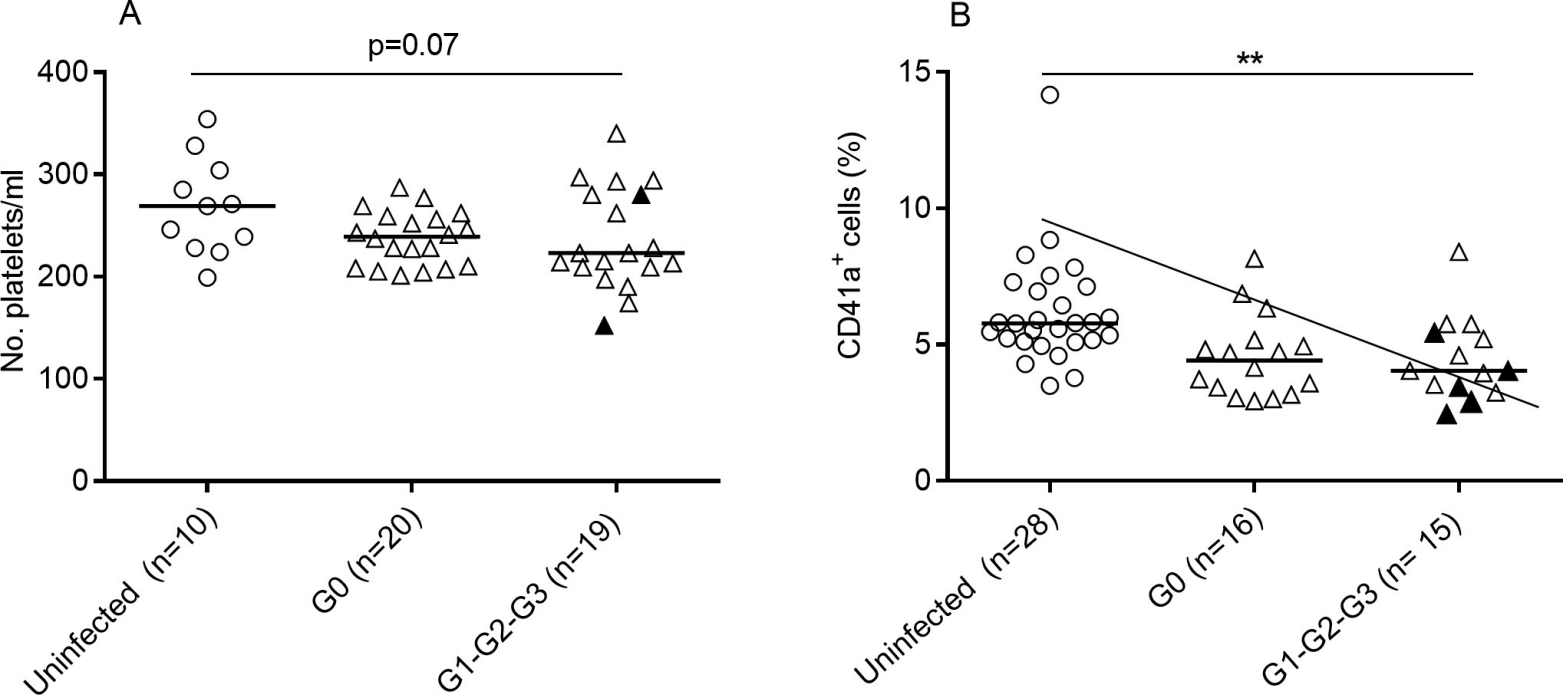
Quantification of platelets in patients with chronic Chagas disease. Platelet levels were measured by platelet counts, using an optical automated hematological counter (Fig 1A) and by the expression of CD41a (Fig 1B) determined by flow cytometry in whole blood samples. Each symbol represents the number of platelets per ml (Fig 1A) or the percentage of CD41a^+^ cells (Fig 1B). Median values are indicated by the horizontal lines. *P ≤ 0.05 and **p ≤ 0.01 calculated by an ANOVA test followed by a Bonferroni test for multiple comparisons. The oblique line indicates a significant tendency between medians calculated using a test for lineal trends. The full-black symbols represent patients belonging to stages G2 and G3.

**Fig 2:**
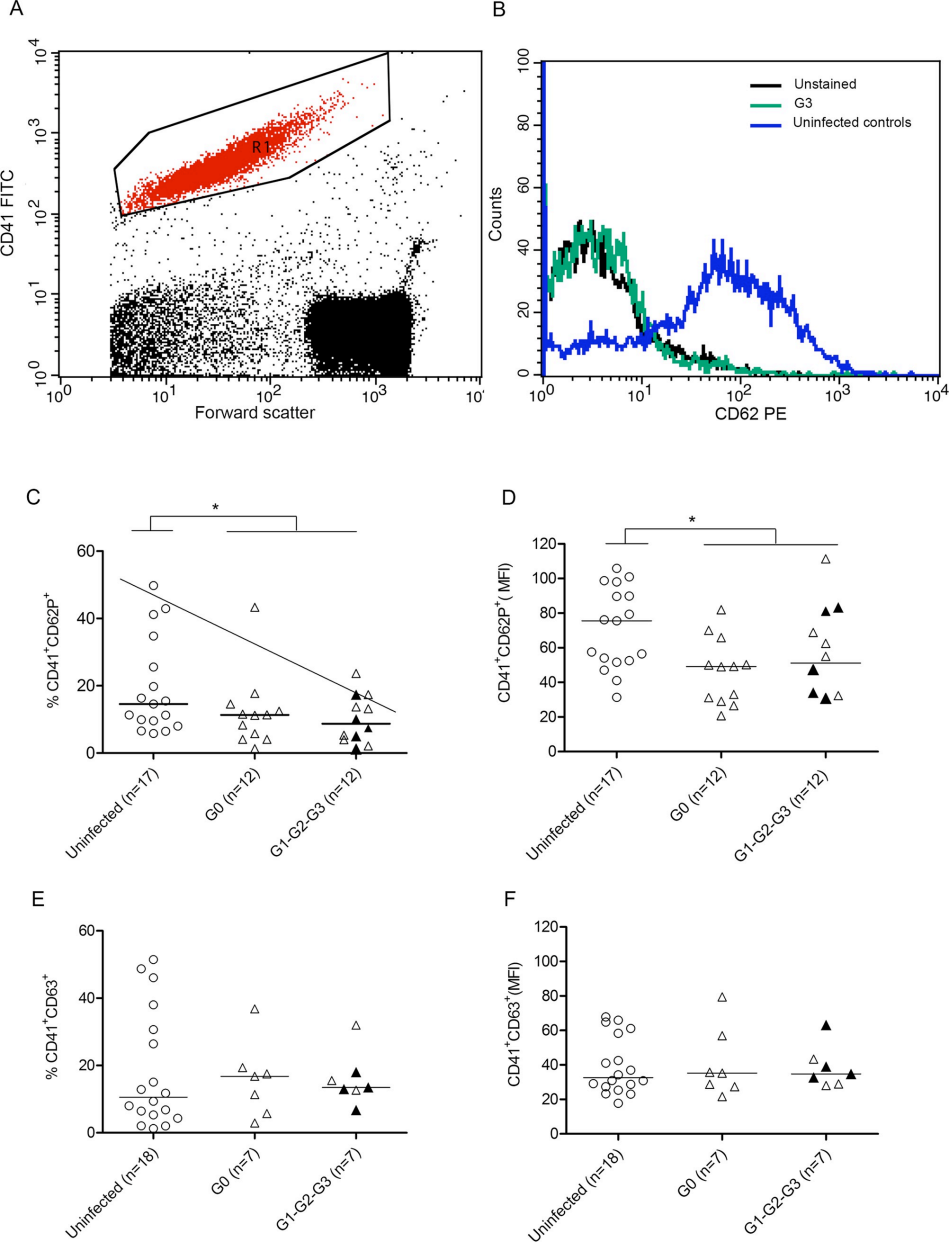
Flow cytometric analysis of activated platelet surfaces. Whole blood was labelled with CD41a, CD62P (P-selectin), CD63 monoclonal antibodies and analyzed by flow cytometry. (A) Platelets were identified by their characteristic side-scatter properties (granularity: x-axis) and positive labeling with a platelet-specific mAb reagent (CD41a-FITC: y-axis). (B) Histogram plots overlaid by unstained, uninfected controls and G3 patient CD62P-PE (x-axis) fluorescence of the events within the “R1” gate were further analyzed. (C) Percentage of CD41a^+^CD62P^+^ and (E) CD41a^+^CD63^+^ cells and the mean fluorescence intensity (MFI) of the (D) CD41a^+^CD62P^+^ and (F) CD41a^+^CD63^+^ expression in each of the chronic Chagas disease patients with different clinical forms of the disease studied. Median values are indicated by the horizontal lines. *P < 0.05 and was calculated by Mann-Whitney test. The oblique line indicates a significant tendency between medians calculated using a test for lineal trends. The symbols in black represent patients belonging to stages G2 and G3.

Platelet levels measured by platelet counts were not altered in patients with chronic Chagas disease compared with uninfected subjects (Fig 1A). Nevertheless, some patients with cardiomyopathy had decreased platelet counts. Likewise, platelet counts measured by the expression of CD41a (Fig 1B) in whole blood, were decreased in patients with chronic Chagas disease compared with those in uninfected healthy subjects, with an inverse association with disease severity (Fig 1B). The CD41a expression per cell, determined by the mean fluorescence intensity (MFI) measurement, was not altered in subjects chronically infected with *T*. *cruzi* compared with that in uninfected healthy controls (S1 Fig). The percentage of platelets (CD41a^+^) that express the activation marker CD62P was diminished in chronically infected subjects, particularly in those subjects with signs of cardiac dysfunction (i.e., G1, G2 and G3 patient groups) [Fig 2A–2C]. Not only was the percentage of CD41a^+^CD62P^+^ cells diminished but also the CD62P MFI decreased in subjects chronically infected with *T*. *cruzi* (Fig 2D). In contrast, no significant differences were observed in the expression of platelet activation marker CD63 (Fig 2E and 2F). Likewise, the expression of P-selectin counter-receptor PSGL-1 was not altered on CD4^+^ and CD8^+^ T cells of Chagas disease patients when compared with that of uninfected healthy subjects (S2 Fig).

### Assessment of platelet function in subjects chronically infected with *T*. *cruzi*

**Fig 3:**
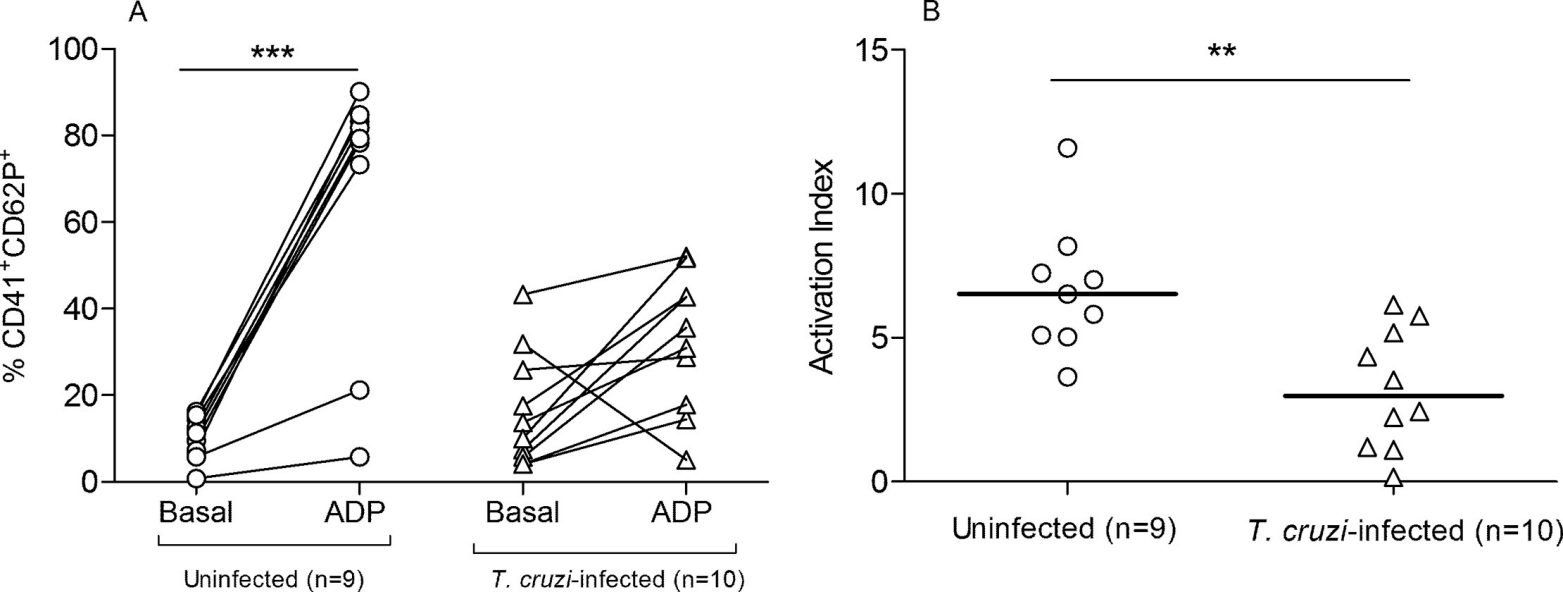
Determination of platelet activation in whole blood of patients with chronic Chagas disease. The percentages of platelets before and after the addition of ADP were measured by flow cytometry. (A) The symbols represent the expression of CD41a^+^CD62P^+^ at the basal levels or after the activation with ADP. ***P <0.001 vs the nonstimulated uninfected healthy group and calculated by using a paired t test. (B) Activation index (expression of CD41a^+^CD62P^+^ cells after activation/basal expression of CD41a^+^CD62P^+^ cells) in each of the subjects evaluated. **P < 0.001 and calculated by using the Mann-Whitney U test.

To assess platelet function, we measured the expression of P-selectin after ADP stimulation in blood samples collected from *T*. *cruzi*-infected and uninfected healthy subjects. The percentage of activated platelets after the stimulation was lower in the *T*. *cruzi*-infected group than that in the uninfected control group (Fig 3A and 3B).

**Fig 4:**
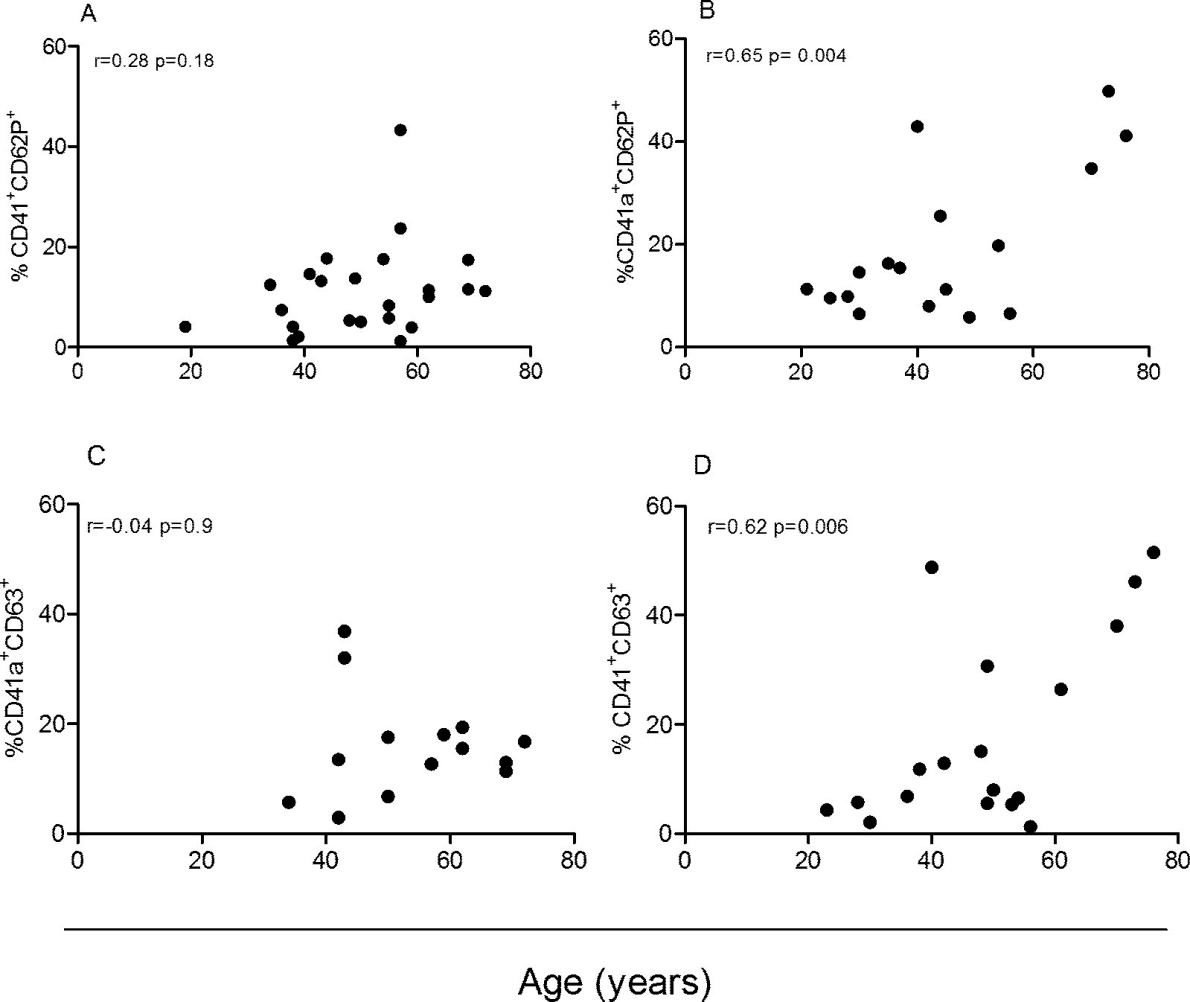
Association between platelet activation and age in patients with chronic Chagas disease and uninfected healthy subjects. Correlation analysis between the levels of activated platelets and age was performed by the Pearson correlation test in *T*. *cruzi*-infected subjects (A, n = 24; C, n = 14) and uninfected healthy subjects (B, n = 17; D, n = 18).

Because several studies have shown that platelet levels mildly decrease with age while platelet activation increases [25], we performed a correlation analysis between age and the frequency of activated platelets in the *T*. *cruzi*-infected and uninfected groups. The frequencies of activated platelets were positively correlated with the age of uninfected healthy subjects (Fig 4B and 4D), but this correlation was abolished in *T*. *cruzi*-infected subjects (Fig 4A and 4C). No significant differences in the percentages of platelet- lymphocyte aggregation were observed between *T*. *cruzi-*infected and uninfected subjects (S3 Fig).

### Measurements of mediators of the coagulation cascade and endothelial activation

**Fig 5:**
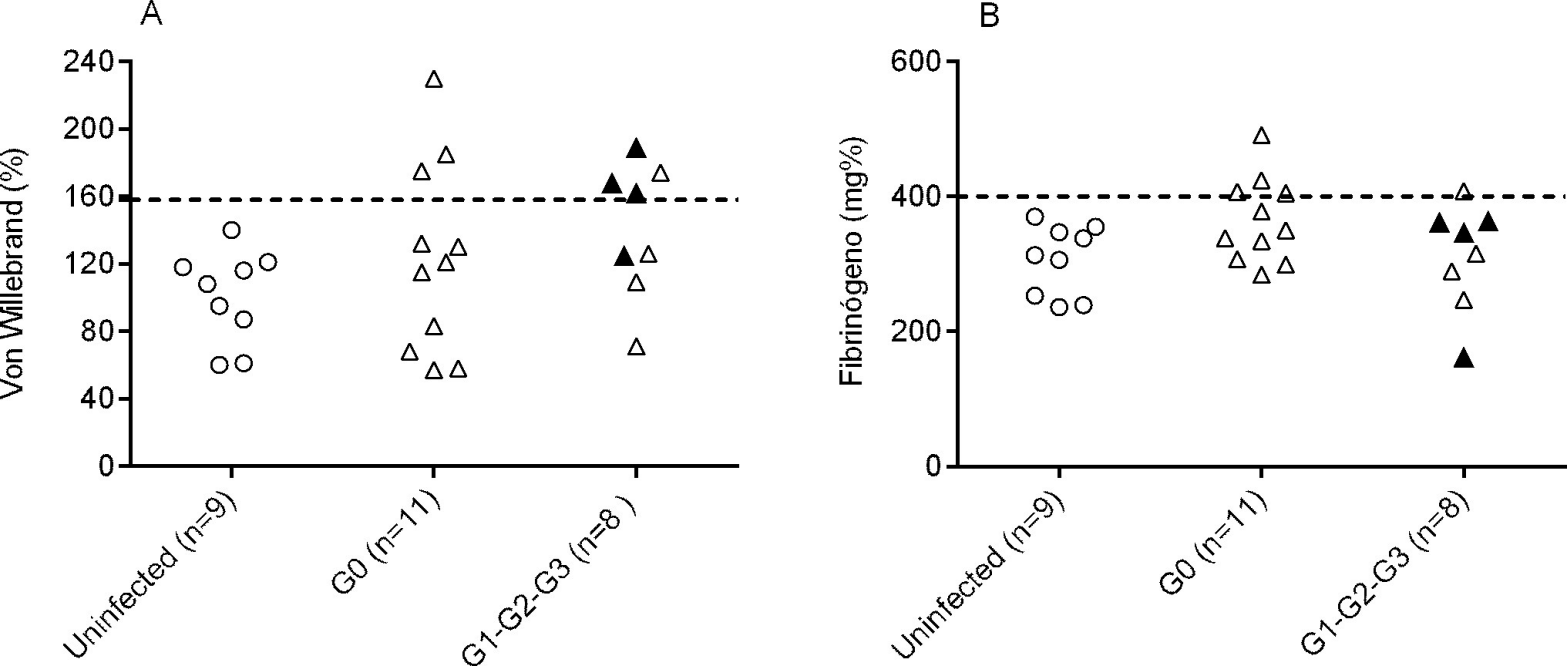
Plasma levels of von Willebrand factor and fibrinogen in patients with chronic Chagas disease. The symbols represent the concentration of (A) von Willebrand factor and (B) fibrinogen for each individual evaluated in the different clinical groups determined. The dotted line shows the maximum reference value (normal range of von Willebrand = 50–160%; normal range of fibrinogen = 200–400 mg/dl). The symbols in black represent patients belonging to stages G2 and G3.

To address whether patients with chronic Chagas disease have alterations in their hemostasis, the von Willebrand and fibrinogen factors were measured. Seven out of 19 patients showed von Willebrand (Fig 5A) levels above the reference concentration while other five patients had increased fibrinogen (Fig 5B) levels. Only one patient showed a concomitant increase in both molecules accounting for a total of 11 out of 19 (58%) patients with at least one molecule altered.

**Fig 6:**
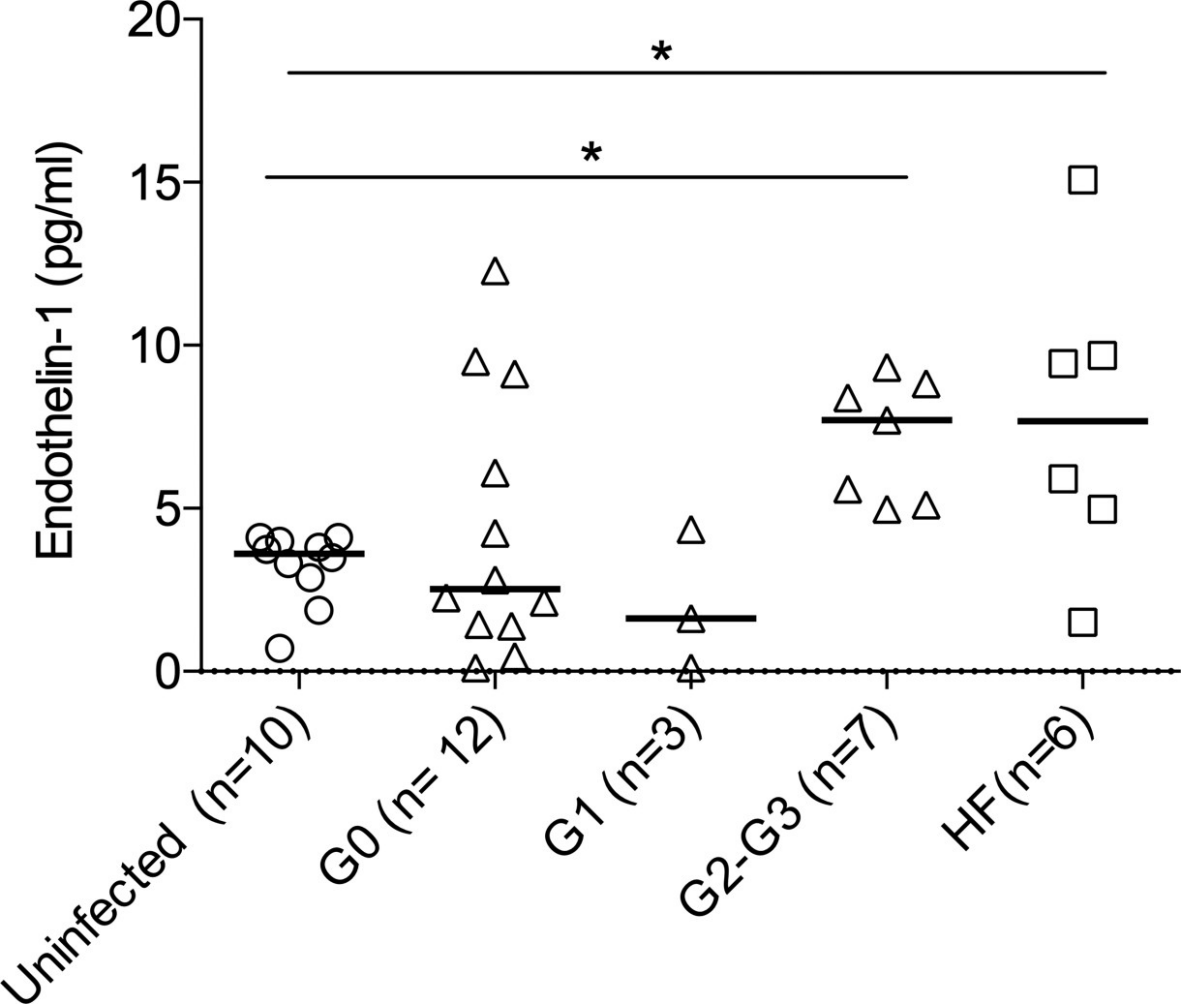
Levels of endothelin-1 in patients with chronic Chagas disease. Plasma endothelin-1 levels were determined by capture ELISA. The symbols represent the concentration of endothelin-1 for each individual evaluated. The horizontal lines indicate the values of the medians for each clinical group established by the Kuschnir classification, as indicated in Materials and Methods. HF, patients with heart failure of origin not related to Chagas disease. *P ≤ 0.05 and was calculated by the unpaired t test.

Increased plasma levels of endothelin-1, which is a marker of endothelial activation, were also found in Chagas disease patients with severe cardiac dysfunction, compared with those in uninfected subjects (Fig 6). Of note, increased endothelin-1 levels in some *T*. *cruzi*-infected patients concurred with increased levels of fibrinogen or the von Willebrand factor and with decreased levels of activated platelets (Table 2).

**Table 2:** Alterations of prothrombotic markers in *T*. *cruzi*-infected patients.

Clinical stage	Endothelin-1 (pg/ml)	Von Willebrand (%)	Fibrinogen (mg%)	CD41a+CD62+ (%)
G0	6.09	185	394	8.32
G0	12.29	115	284	17.71
G0	7.76	58	424	14.57
G0	9.50	132	491	ND
G1	9.13	174	315	13.19

Note. The values in healthy individuals are the following: Endothelin-1, median ± SD = 3.2 ± 1.11 (pg/ml); von Willebrand (VB), range = 50–160%; Fibrinogen, range = 200–400 mg%; and CD41a^+^CD62^+^, median ± SD = 34 ± 13%. Patients 1–5 displayed increased endothelin-1 levels.

**Fig 7:**
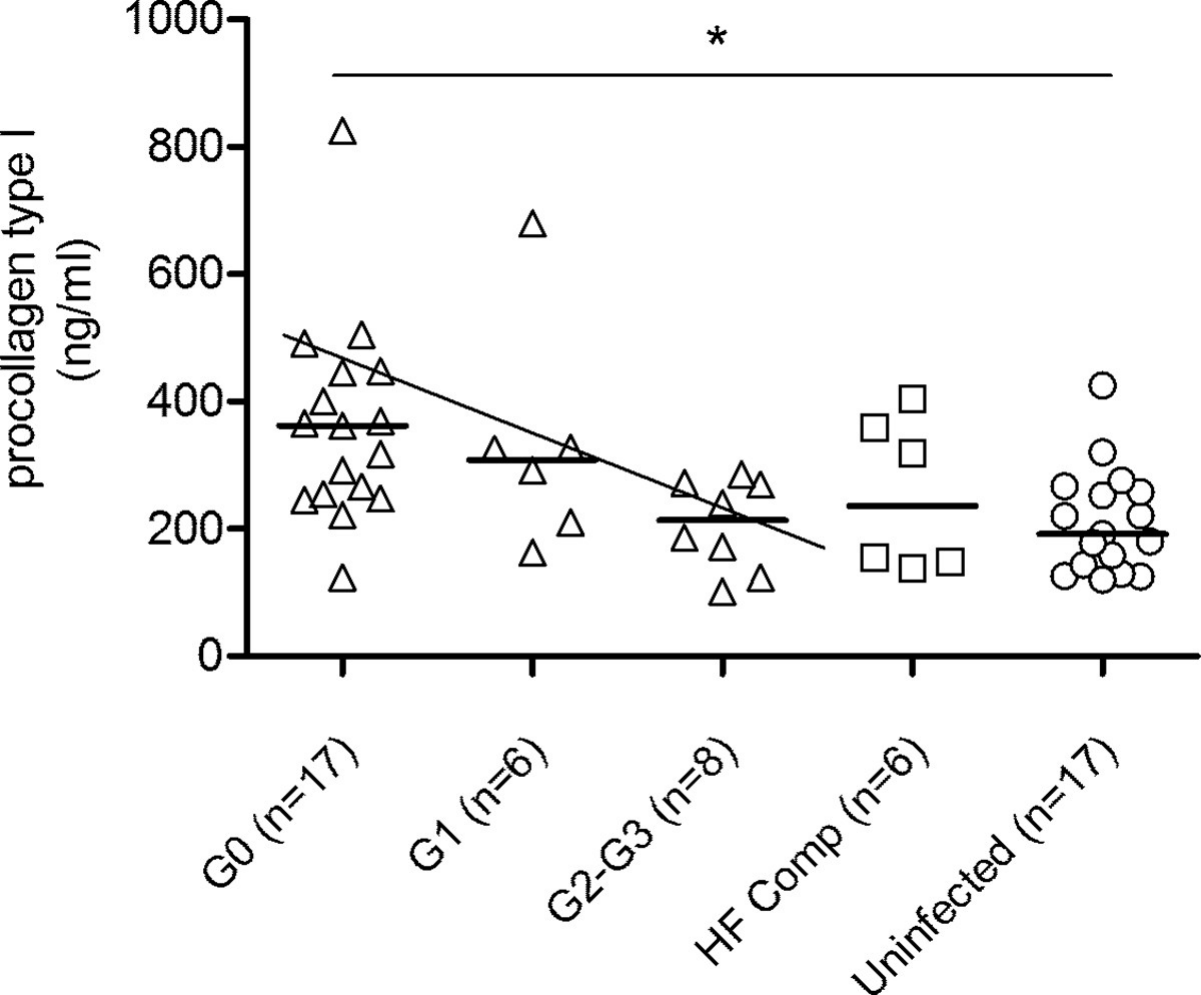
Procollagen levels in patients with chronic Chagas disease. The levels of procollagen peptides were measured by capture ELISA. The symbols represent the concentration of procollagen for each individual evaluated in the different clinical groups determined by the Kuschnir classification, as indicated in Materials and Methods. The horizontal lines show the median. HF, patients with heart failure unrelated to Chagas disease. *P ≤ 0.05 and was calculated by the unpaired t test. The oblique line indicates a significant tendency between medians using a test for lineal trends; P = 0.0006.

Serum levels of the tissue remodeling factor procollagen I C-terminal propeptide inversely correlated with the severity of the cardiac disease in *T*. *cruzi*-infected subjects (Fig 7). The level of endothelial activation and tissue remodeling was also evaluated in a group of patients with heart failure of noninfectious etiology. The levels of endothelin-1 and procollagen 1 in these patients were similar to those found in *T*. *cruzi*-infected subjects with severe cardiac disease (Figs 6 and 7).

## Discussion

Platelets, which participate in thrombotic processes, have different roles in vascular biology and in the immune response, including protective functions and other functions that would contribute to an adverse inflammatory state [26]. Our results showed alterations in the levels and function of platelets, tissue remodeling and endothelial activation in chronically infected patients, even in those patients who do not yet present with cardiac symptoms. The negative trend between the percentages of platelets and disease severity might be explained either by a platelet activation with shortening of survival or by impaired platelet production in the bone marrow. P-selectin (CD62P) is a protein that mediates adhesion of leukocytes to the endothelium and in the aggregation of platelets. After being activated by agonist including thrombin, collagen and adenosine diphosphate), the molecules stored in the alpha-granules in platelets or in the Weibel-Palade bodies in endothelial cells are exposed in the cell surface [16,27].

We have previously shown that soluble P-selectin levels are increased in the circulation of patients with chronic Chagas disease [3], supporting the idea that increased platelet activation might in turn induce the release of P-selectin in a way that regulates exacerbated platelet activation [9,28]. The decreased levels of P-selectin-expressing platelets in patients with signs of cardiac dysfunction observed in this study are in agreement with the higher levels of soluble P-selectin observed in more severe stages of chronic Chagas disease [3]. In contrast, the expression of the activation molecule CD63 was similar between chronic chagasic patients and uninfected control, which could be due to the fact that it is a molecule that is not preformed as P-selectin and is not cleaved following activation [29].

Platelet loss is a common feature in viral [30] and bacterial infections [31,32]. Here, we provide further support that such a loss might also occur in parasitic infections. Supporting the relationship between infection and platelet loss is the observation of a positive association between platelet activation and age in uninfected subjects, an association that is lost in *T*. *cruzi*-infected subjects. Although activated platelets can contribute to an increased risk of thrombotic events [33], they might also be important in host defense. Thus, upon activation, platelets release a range of chemokines that attract and activate leukocytes and concurrently, various platelet surface molecules (i.e., P-selectin and GPIIb/IIIa). The proinflammatory cytokines IL-1 and TGF-beta mediate the crosstalk with endothelial cells and leukocytes in order to activate them and facilitate transendothelial migration at sites of injury or inflammation [34–39]. Platelets might also release superoxide, peroxide and hydroxyl radicals upon activation [40] and exert phagocytotic activity [41,42]. Here, we demonstrate that not only the frequencies of platelets but also their functions are impaired in subjects chronically infected with *T*. *cruzi*. Of note, we did not find increased platelet-lymphocyte aggregation more likely in line with the gradually impairment of the immune response in the chronic phase rather than a pro-thrombotic state of chronically *T*. *cruzi*-infected subjects.

In our study, we found that 11 out of 19 patients had increased levels of the von Willebrand factor or fibrinogen and that these levels were not restricted to the most severe forms of the disease. In contrast, we did find increased levels of endothelin-1 in the more severe forms of chronic Chagas disease, which was consistent with the findings in uninfected subjects suffering from heart failure, who are known to present with neurohormonal activation and a high degree of endothelial injury [43,44]. Of note, some patients in the asymptomatic stage also had increased levels of endothelin-1 in association with increased levels of the von Willebrand factor which is synthesized and stored not only in megakaryocytes but also in endothelial cells. Although it is possible that this increase reflects a state of greater immune activation as a consequence of the release of mediators that activate the endothelium, we cannot rule out an increased risk of progression in these patients. High endothelial activation in chronically *T*. *cruzi*-infected subjects is also supported by increased soluble VCAM-1 (vascular cell adhesion protein 1 molecule) levels observed in these patients [3]. Of note, concomitant increases of fibrinogen and the von Willebrand factor was hardly observed supporting the idea that various coagulation pathways can be altered in different patients with chronic Chagas disease. The von Willebrand factor might participate in both primary and secondary hemostasis [45].

Other authors have evaluated a group of hypercoagulable biomarkers in patients with chronic Chagas disease, demonstrating that fragments of prothrombin 1 + 2, endogenous potential thrombin, the plasmin-antiplasmin complex (PAP) and soluble P-selectin levels were increased in subjects chronically infected with *T*. *cruzi* [11,46–48] and that these alterations decreased after a specific treatment with benznidazole [11,48]. In the experimental infection in mice, an increase in the levels of thromboxane A2 and endothelin-1 associated with enhanced platelet adherence and aggregation was also reported [49,50].

The higher levels of collagen I propeptides in infected subjects with less severe forms of the disease might be related to the homeostatic process of remodeling as a consequence of the immune response. Fibrosis, which is the consequence of a wrong process of tissue remodeling, can arise if the damage persists, since inflammation can change the triggers from a reparative response to a profibrotic harmful response in an attempt to continue repair [51]. Therefore, under pathological conditions, the composition and amount of collagens change [52,53]. It is known that patients with decompensated heart failure have elevated levels of collagen I propeptides as a result of an acute tissue injury and that the levels return to normal values in the compensated stage [54], which is in agreement with our findings presented in this study. Tissue remodeling in the more severe stages of chronic Chagas disease likely involved the participation of other collagen types. Importantly, an increased ratio of collagen types I, III and V vs collagen type IV, rather than their absolute amount, determines epithelial and endothelial cell dysfunction in liver fibrosis [55]. Moreover, collagens play a direct role in hemostasis by their interactions with platelet receptors, contributing to platelet spreading and activation [56], which might also change in the chronic phase of *T*. *cruzi* infection.

Although the alterations observed in the different markers evaluated could be interpreted as a state of greater risk to thrombotic processes, it is likely that these are indicators of a sustained immune response. In previous studies, we have shown that the persistence of *T*. *cruzi* is able to induce a process of functional depletion of T lymphocytes that is more pronounced as the disease progresses [57]. The findings in the present study, support that platelets dysfunction, endothelial activation and altered tissue remodeling are induced by persistent infection with *T*. *cruzi*.

## Supporting information

S1 Fig Expression of CD41a in platelets of patients with chronic Chagas disease. Whole blood was stained with the CD41a monoclonal antibody and analyzed by flow cytometry. Each symbol represents the Mean Fluoresce Intensity (MFI) of CD41a^+^ cells. The horizontal lines indicate the values of the medians for each clinical group established by the Kuschnir classification, as indicated in Materials and Methods. The symbols in black represent patients belonging to clinical stages G2 and G3.
(TIF)

S2 Fig Expression of PSGL-1 in patients with chronic Chagas disease. A whole blood labeling was made in citrate using specific antibodies for CD4, CD8 and PSGL-1, and the samples were analyzed by flow cytometry. Each symbol represents the percentage of CD4 (A) or CD8 (B) lymphocytes expressing PSGL-1 (left panel A and B) and the mean expression per cell of PSGL-1 (right panel A and B) for each individual evaluated. The horizontal lines indicate the values of the medians for each clinical group established by the Kuschnir classification, as indicated in Materials and Methods.
(TIF)

S3 Fig Analysis of platelet-lymphocytes aggregation in chronic Chagas disease patients. Whole blood was stained with the CD41a and CD45 monoclonal antibodies and analyzed by flow cytometry. Lymphocytes were identified based on cell size and granularity using the forward and side scatter and the percentages of CD45 vs. CD41a were determined. The horizontal lines indicate the values of the medians for each group.
(TIF)

S1 File Raw data of the manuscript. (XLSX)

## References

[1] WHO. Chagas disease (American trypanosomiasis) World Health Organization 2015;

[2] Dutra WO, Martins-Filho OA, Cançado JR, Pinto-Dias JC, Brener Z, Freeman Júnior GL, et al Activated T and B lymphocytes in peripheral blood of patients with Chagas’ disease. Int Immunol. 1994;6: 499–506. 10.1093/intimm/6.4.499 8018591

[3] Laucella S, De Titto EH, Segura EL, Orn A, Rottenberg ME. Soluble cell adhesion molecules in human Chagas’ disease: association with disease severity and stage of infection. Am J Trop Med Hyg. 1996;55: 629–34. 10.4269/ajtmh.1996.55.629 9025689

[4] Albareda MC, Laucella SA, Alvarez MG, Armenti AH, Bertochi G, Tarleton RL, et al Trypanosoma cruzi modulates the profile of memory CD8+ T cells in chronic Chagas’ disease patients. Int Immunol. 2006;18: 465–471. 10.1093/intimm/dxh387 16431876

[5] Laucella SA, Mazliah DP, Bertocchi G, Alvarez MG, Cooley G, Viotti R, et al Changes in Trypanosoma cruzi-specific immune responses after treatment: surrogate markers of treatment efficacy. Clin Infect Dis. 2009;49: 1675–84. 10.1086/648072 19877967 PMC2805187

[6] Chimen M, Apta BHR, Mcgettrick HM. Introduction: T Cell Trafficking in Inflammation and Immunity. Methods Mol Biol. 2017;1591: 73–84. 10.1007/978-1-4939-6931-9_6 28349476

[7] Weller A, Isenmann S, Vestweber D. Cloning of the mouse endothelial selectins. Expression of both E- and P-selectin is inducible by tumor necrosis factor alpha. J Biol Chem. 1992;267: 15176–83. 1378846

[8] Yao L, Pan J, Setiadi H, Patel KD, McEver RP. Interleukin 4 or oncostatin M induces a prolonged increase in P-selectin mRNA and protein in human endothelial cells. J Exp Med. 1996;184: 81–92. 10.1084/jem.184.1.81 8691152 PMC2192668

[9] Khew-Goodall Y, Butcher CM, Litwin MS, Newlands S, Korpelainen EI, Noack LM, et al Chronic expression of P-selectin on endothelial cells stimulated by the T-cell cytokine, interleukin-3. Blood. 1996;87: 1432–8. 8608233

[10] Ley K, Kansas GS. Selectins in T-cell recruitment to non-lymphoid tissues and sites of inflammation. Nat Rev Immunol. 2004;4: 325–35. 10.1038/nri1351 15122198

[11] Laucella SA, Segura EL, Riarte A, Sosa ES. Soluble platelet selectin (sP-selectin) and soluble vascular cell adhesion molecule-1 (sVCAM-1) decrease during therapy with benznidazole in children with indeterminate form of Chagas’ disease. Clin Exp Immunol. 1999;118: 423–7. 10.1046/j.1365-2249.1999.01070.x 10594562 PMC1905450

[12] Marin-Neto JA, Simões MV, Rassi Junior A. Pathogenesis of chronic Chagas cardiomyopathy: the role of coronary microvascular derangements. Rev Soc Bras Med Trop. 46: 536–41. 10.1590/0037-8682-0028-2013 23904079

[13] Tanowitz HB, Huang H, Jelicks LA, Chandra M, Loredo ML, Weiss LM, et al Role of endothelin 1 in the pathogenesis of chronic chagasic heart disease. Infect Immun. 2005;73: 2496–503. 10.1128/IAI.73.4.2496-2503.2005 15784596 PMC1087455

[14] Pinazo M-J, Tàssies D, Muñoz J, Fisa R, Posada E de J, Monteagudo J, et al Hypercoagulability biomarkers in Trypanosoma cruzi -infected patients. Thromb Haemost. 2011;106: 617–23. 10.1160/TH11-04-0251 21866301

[15] Freeman BD, Machado FS, Tanowitz HB, Desruisseaux MS. Endothelin-1 and its role in the pathogenesis of infectious diseases. Life Sciences. 2014 pp. 110–119. 10.1016/j.lfs.2014.04.021 24780317 PMC4538933

[16] Semple JW, Italiano JE, Freedman J. Platelets and the immune continuum. Nat Rev Immunol. 2011;11: 264–74. 10.1038/nri2956 21436837

[17] Männel DN, Grau GE. Role of platelet adhesion in homeostasis and immunopathology. Mol Pathol. 1997;50: 175–85. 10.1136/mp.50.4.175 9350300 PMC379623

[18] von Hundelshausen P, Koenen RR, Weber C. Platelet-mediated enhancement of leukocyte adhesion. Microcirculation. 2009;16: 84–96. 10.1080/10739680802564787 19115139

[19] TDR/WHO. Research Priorities for Chagas Disease, Human African Trypanosomiasis and Leishmaniasis. WHO Tech Rep Ser. 2012; 23484340

[20] Kuschnir E, Sgammini H, Castro R, Evequoz C, Ledesma R, Brunetto J. [Evaluation of cardiac function by radioisotopic angiography, in patients with chronic Chagas cardiopathy]. Arq Bras Cardiol. 1985;45: 249–56. 3835868

[21] Viotti R, Vigliano C, Alvarez MG, Lococo B, Petti M, Bertocchi G, et al Impact of aetiological treatment on conventional and multiplex serology in chronic Chagas disease. PLoS Negl Trop Dis. 2011;5: e1314 10.1371/journal.pntd.0001314 21909451 PMC3167788

[22] CLAUSS A. [Rapid physiological coagulation method in determination of fibrinogen]. Acta Haematol. 1957;17: 237–46. 10.1159/000205234 13434757

[23] Huang G-S, Hu M-H, Lee C-H, Tsai C-S, Lin T-C, Li C-Y. Can hypertonic saline influence platelet P selectin expression and platelet-leukocyte aggregation? Am J Emerg Med. 2010;28: 37–43. 10.1016/j.ajem.2008.09.026 20006199

[24] Bath PM, May J, Heptinstall S. Clinical utility of remote platelet function measurement using P-selectin: assessment of aspirin, clopidogrel, and prasugrel and bleeding disorders. Platelets. 2018;29: 425–430. 10.1080/09537104.2018.1445839 29667460

[25] Sepúlveda C, Palomo I, Fuentes E. Primary and secondary haemostasis changes related to aging. Mech Ageing Dev. 2015;150: 46–54. 10.1016/j.mad.2015.08.006 26296601

[26] Kamel MM, Fouad SA, Basyoni MMA. P selectins and immunological profiles in HCV and Schistosoma mansoni induced chronic liver disease. BMC Gastroenterol. 2014;14: 132 10.1186/1471-230X-14-132 25066324 PMC4119237

[27] Peeters CFJM Ruers TJM, Westphal JR, de Waal RMW. Progressive loss of endothelial P-selectin expression with increasing malignancy in colorectal cancer. Lab Invest. 2005;85: 248–56. 10.1038/labinvest.3700217 15640834

[28] Larsen E, Celi A, Gilbert GE, Furie BC, Erban JK, Bonfanti R, et al PADGEM protein: a receptor that mediates the interaction of activated platelets with neutrophils and monocytes. Cell. 1989;59: 305–12. 2478294 10.1016/0092-8674(89)90292-4

[29] Pols MS, Klumperman J. Trafficking and function of the tetraspanin CD63. Experimental Cell Research. 2009 10.1016/j.yexcr.2008.09.020 18930046

[30] Passos AM, Treitinger A, Spada C. An overview of the mechanisms of HIV-related thrombocytopenia. Acta Haematol. 2010;124: 13–8. 10.1159/000313782 20606410

[31] Kraemer BF, Campbell RA, Schwertz H, Franks ZG, Vieira de Abreu A, Grundler K, et al Bacteria differentially induce degradation of Bcl-xL, a survival protein, by human platelets. Blood. 2012;120: 5014–20. 10.1182/blood-2012-04-420661 23086749 PMC3525025

[32] Towhid ST, Nega M, Schmidt E-M, Schmid E, Albrecht T, Münzer P, et al Stimulation of platelet apoptosis by peptidoglycan from Staphylococcus aureus 113. Apoptosis. 2012;17: 998–1008. 10.1007/s10495-012-0718-1 22752708

[33] Mayne E, Funderburg NT, Sieg SF, Asaad R, Kalinowska M, Rodriguez B, et al Increased platelet and microparticle activation in HIV infection: upregulation of P-selectin and tissue factor expression. J Acquir Immune Defic Syndr. 2012;59: 340–6. 10.1097/QAI.0b013e3182439355 22156911 PMC3299881

[34] von Hundelshausen P, Weber C. Platelets as immune cells: bridging inflammation and cardiovascular disease. Circ Res. 2007;100: 27–40. 10.1161/01.RES.0000252802.25497.b7 17204662

[35] Henn V, Slupsky JR, Gräfe M, Anagnostopoulos I, Förster R, Müller-Berghaus G, et al CD40 ligand on activated platelets triggers an inflammatory reaction of endothelial cells. Nature. 1998;391: 591–4. 10.1038/35393 9468137

[36] Santoso S, Sachs UJH, Kroll H, Linder M, Ruf A, Preissner KT, et al The junctional adhesion molecule 3 (JAM-3) on human platelets is a counterreceptor for the leukocyte integrin Mac-1. J Exp Med. 2002;196: 679–91. 10.1084/jem.20020267 12208882 PMC2194005

[37] Maugeri N, Rovere-Querini P, Evangelista V, Covino C, Capobianco A, Bertilaccio MTS, et al Neutrophils phagocytose activated platelets in vivo: a phosphatidylserine, P-selectin, and {beta}2 integrin-dependent cell clearance program. Blood. 2009;113: 5254–65. 10.1182/blood-2008-09-180794 19264679

[38] Lievens D, von Hundelshausen P. Platelets in atherosclerosis. Thromb Haemost. 2011;106: 827–38. 10.1160/TH11-08-0592 22012554

[39] Lam FW, Burns AR, Smith CW, Rumbaut RE. Platelets enhance neutrophil transendothelial migration via P-selectin glycoprotein ligand-1. Am J Physiol Heart Circ Physiol. 2011;300: H468–75. 10.1152/ajpheart.00491.2010 21169400 PMC3044064

[40] Martin-Ventura JL, Madrigal-Matute J, Martinez-Pinna R, Ramos-Mozo P, Blanco-Colio LM, Moreno JA, et al Erythrocytes, leukocytes and platelets as a source of oxidative stress in chronic vascular diseases: detoxifying mechanisms and potential therapeutic options. Thromb Haemost. 2012;108: 435–42. 10.1160/TH12-04-0248 22836558

[41] Zucker-Franklin D, Seremetis S, Zheng ZY. Internalization of human immunodeficiency virus type I and other retroviruses by megakaryocytes and platelets. Blood. 1990;75: 1920–3. 2337668

[42] Youssefian T, Drouin A, Massé J-M, Guichard J, Cramer EM. Host defense role of platelets: engulfment of HIV and Staphylococcus aureus occurs in a specific subcellular compartment and is enhanced by platelet activation. Blood. 2002;99: 4021–9. 10.1182/blood-2001-12-0191 12010803

[43] Abukar Y, May CN, Ramchandra R. Role of endothelin-1 in mediating changes in cardiac sympathetic nerve activity in heart failure. Am J Physiol Regul Integr Comp Physiol. 2016;310: R94–9. 10.1152/ajpregu.00205.2015 26468257

[44] Chai SB, Li XM, Pang YZ, Qi YF, Tang CS. Increased plasma levels of endothelin-1 and urotensin-II in patients with coronary heart disease. Heart Vessels. 2010;25: 138–43. 10.1007/s00380-009-1178-6 20339975

[45] Palta S, Saroa R, Palta A. Overview of the coagulation system. Indian Journal of Anaesthesia. 2014 10.4103/0019-5049.144643 25535411 PMC4260295

[46] Salomone OA, Caeiro TF, Madoery RJ, Amuchástegui M, Omelinauk M, Juri D, et al High plasma immunoreactive endothelin levels in patients with Chagas’ cardiomyopathy. Am J Cardiol. 2001;87: 1217–20; A7. 11356406 10.1016/s0002-9149(01)01502-8

[47] Herrera RN, Díaz E, Pérez Aguilar R, Bianchi J, Berman S, Luciardi HL. [Prothrombotic state in early stages of chronic Chagas’ disease. Its association with thrombotic risk factors]. Arch Cardiol Mex. 75 Suppl 3: S3-38–48. 16366168

[48] Pinazo M-J, Posada E de J, Izquierdo L, Tassies D, Marques A-F, de Lazzari E, et al Altered Hypercoagulability Factors in Patients with Chronic Chagas Disease: Potential Biomarkers of Therapeutic Response. PLoS Negl Trop Dis. 2016;10: e0004269 10.1371/journal.pntd.0004269 26727000 PMC4700971

[49] Tanowitz HB, Burns ER, Sinha AK, Kahn NN, Morris SA, Factor SM, et al Enhanced platelet adherence and aggregation in Chagas’ disease: a potential pathogenic mechanism for cardiomyopathy. Am J Trop Med Hyg. 1990;43: 274–81. 10.4269/ajtmh.1990.43.274 2121055

[50] Prado CM, Jelicks LA, Weiss LM, Factor SM, Tanowitz HB, Rossi MA. The vasculature in chagas disease. Adv Parasitol. 2011; 10.1016/B978-0-12-385895-5.00004-9 PMC3557505 21884888

[51] Karsdal MA, Nielsen SH, Leeming DJ, Langholm LL, Nielsen MJ, Manon-Jensen T, et al The good and the bad collagens of fibrosis—Their role in signaling and organ function. Adv Drug Deliv Rev. 2017;121: 43–56. 10.1016/j.addr.2017.07.014 28736303

[52] Leeming DJ, Byrjalsen I, Jiménez W, Christiansen C, Karsdal MA. Protein fingerprinting of the extracellular matrix remodelling in a rat model of liver fibrosis—a serological evaluation. Liver Int. 2013;33: 439–47. 10.1111/liv.12044 23279004

[53] Karsdal MA, Henriksen K, Leeming DJ, Woodworth T, Vassiliadis E, Bay-Jensen A-C. Novel combinations of Post-Translational Modification (PTM) neo-epitopes provide tissue-specific biochemical markers—are they the cause or the consequence of the disease? Clin Biochem. 2010;43: 793–804. 10.1016/j.clinbiochem.2010.03.015 20381482

[54] Ruiz-Ruiz FJ, Ruiz-Laiglesia FJ, Samperiz-Legarre P, Lasierra-Diaz P, Flamarique-Pascual A, Morales-Rull JL, et al Propeptide of procollagen type I (PIP) and outcomes in decompensated heart failure. Eur J Intern Med. 2007;18: 129–34. 10.1016/j.ejim.2006.09.014 17338965

[55] Baiocchini A, Montaldo C, Conigliaro A, Grimaldi A, Correani V, Mura F, et al Extracellular Matrix Molecular Remodeling in Human Liver Fibrosis Evolution. PLoS One. 2016;11: e0151736 10.1371/journal.pone.0151736 26998606 PMC4801190

[56] Manon-Jensen T, Kjeld NG, Karsdal MA. Collagen-mediated hemostasis. J Thromb Haemost. 2016;14: 438–48. 10.1111/jth.13249 26749406

[57] Albareda MC, Laucella SA. Modulation of Trypanosoma cruzi-specific T-cell responses after chemotherapy for chronic Chagas disease. Mem Inst Oswaldo Cruz. Instituto Oswaldo Cruz, Ministério da Saúde; 2015;110: 414–21. 10.1590/0074-02760140386 25993507 PMC4489479

